# A multi-center, randomized, controlled trial to assess the efficacy of optimization of drug prescribing in an elderly population, at 18 months of follow-up, in the evolution of functional autonomy: the OPTIM study protocol

**DOI:** 10.1186/s12877-017-0600-7

**Published:** 2017-08-30

**Authors:** Virginie Dauphinot, Elodie Jean-Bart, Pierre Krolak-Salmon, Christelle Mouchoux

**Affiliations:** 1 Memory Research Centre of Lyon (CMRR); Geriatrics Unit, Charpennes Hospital, University Hospital of Lyon, Villeurbanne, France; 2 Research Clinic Centre (CRC) - VCF (Aging – Brain - Frailty), Charpennes Hospital, University Hospital of Lyon, Villeurbanne, France; 3 Pharmacy department, Charpennes Hospital, University Hospital of Lyon, Villeurbanne, France; 4 University Lyon 1, INSERM, U1028; UMR CNRS 5292, Research Centre of Neurosciences of Lyon, Lyon, France; 5 University Lyon 1, ISPB, Pharmacie Clinique, Pharmacocinétique et Évaluation du Médicament, Lyon, France; 6 Hôpital des Charpennes, 27 rue Gabriel Péri, 69100 Villeurbanne, France

**Keywords:** Randomized controlled trial, Inappropriate prescribing, Elderly, Daily living activities, Neurocognitive disorders

## Abstract

**Background:**

Pharmacotherapy is necessary for the management of many diseases which number increased with aging. However, potentially inappropriate prescriptions and polymedication increases iatrogenic risks and can lead to adverse events. To limit the consequences of potentially harmful prescriptions, optimization of drug prescribing is a major stake of improving quality and safety of care in the elderly. The purpose of the OPTIM study is to study the impact of the optimization of drug prescribing on the evolution of functional autonomy at 18 months of follow-up.

**Methods:**

A multicenter, open-label, Randomized Controlled Trial was designed to assess the impact of an optimization program of drug prescribing consisting in a clinical medication review by a pharmacist, in collaboration with specialist physician of the geriatric/memory center and the referent physician, on the evolution of functional autonomy level, measured during 18 months of follow-up. The study will include 302 elderly outpatients visiting geriatric and memory centers, randomly distributed in one of the two parallel groups. One group will benefit of the intervention, while the other will be considered as control group. The effect of the intervention on evolution of the level of autonomy function, defined with repeated measures, will be estimated in a generalized linear mixed model. The intervention will be considered significant if the interaction between time and the study group is significant. Secondary analysis will be conducted to assess the impact of the intervention on secondary clinical outcomes.

**Discussion:**

The “OPTIM” program should enable optimization of drug prescribing in elderly patients and therefore slow or prevent progression to loss of functional autonomy. It should also help to strengthen collaboration between the hospital team of geriatric/neurologist, the pharmacist and the private practice who are all involved in caring for the patient’s health. The benefits for the patient are thus optimizing its medical management by linking health professionals met during his care pathway. In addition, pharmaceutical recommendations sent to referent physicians should help raise awareness of the prescription of drugs in these patients.

**Trial registration number clinicaltrials:**

NCT02740764

## Background

Drug prescription is a fundamental component of the care of elderly people and its optimization has become a major public-health field worldwide [1]. However, several age-related characteristics increase the iatrogenic risk including reduced physiological reserve and homeostasis, pharmacodynamics and pharmacokinetic modifications, acute and chronic diseases and polypharmacy [2–4]. In addition, with an increased frequency of polymedication among elderly people, the proportion of interaction effects between drugs may be more frequent, and can conduct themselves to increased adverse events [5, 6]. Some age-related syndromes, especially cognitive impairment and loss of functional autonomy, may also affect the medication efficacy and the ability of elderly people to take their medication. Indeed, these characteristics of ageing and geriatric syndromes have an impact on medication prescribing for elderly people and represent a challenging process in the selection of appropriate pharmacotherapy. The diversity of acute medical conditions of elderly people makes generalization of prescribing decisions difficult for clinicians. Therefore, the medication prescribing in elderly people is too often inappropriate. Different types of potentially inappropriate prescriptions (PIP) have been described: the ‘overuse’ (use of drugs with risks greater than the benefits and prescription of drugs without valid indication); the ‘underuse’ (absence of prescription of potentially beneficial drugs); and the ‘misuse’ (use of inappropriate dosage, duration, administration modalities, drug prescription with clinically significant drug–drug and drug–disease interactions, redundancy, cost). PIP are frequent in the elderly in outpatients as well as in patients living in health facility with a prevalence from 19% to 54% [7–10]. A previous study showed that 42% of elderly have at least one prescription drug without a valid indication [11]. This study also showed that the dosage and duration of treatment were inappropriate for half of the patients. The most concerned drugs were those used in cardiovascular therapies, analgesics, hypoglycaemic agents, psychotropics and anticoagulants [12–14]. Besides, many diseases remain poorly controlled in elderly e.g. drugs in heart failure and osteoporosis are under-used by 20 to 70% of patients [15, 16]. The PIP has been found associated with an increased risk of morbidity, adverse drug events (ADE), hospitalization, mortality and increased healthcare costs [17–19]. In terms of use of the health care system, problems related to misuse of drugs are common since about 20% of emergency use in elderly patients were found associated to an adverse event related to drugs, in Italy and in the United States [20, 21]. In France, proportion of hospitalisations due to ADE was estimated at 3.6% in 2007 [22]. However, a study conducted in the United States has shown that nearly 28% of adverse events related to drug prescriptions could be avoided [23]. Besides, there is evidence for harmful effects associated with the polypharmacy and the use of specific classes of drugs. Various studies have shown that cumulative anticholinergic and sedative exposure was associated with functional and cognitive impairment in elderly people [24–27]. In a longitudinal study conducted among inpatients, prescriptions of drugs with anticholinergic and sedative effect were associated with increased risk of in-hospital fall [28].

Since drug consumption in older people is high, many commonly prescribed drugs have anticholinergic or sedative effects (such as antiemetics, antispasmodics, antiarythmic drugs, antihypertensives, analgesics, psychotropic drugs, antihistaminics and bronchodilatators). An estimated 7,5% of community-dwelling older patients use anticholinergic drugs [29]. Therefore reducing the number and dose of anticholinergic and sedative medication may reduce the impact on cognition and slow functional dependence progression.

To limit the consequences of potentially harmful prescriptions, optimizing drug therapy is a major focus of improving quality and safety of care in the elderly. In a multidisciplinary team of healthcare providers, various pharmacist interventions can be implemented throughout the whole care process. The medication reviews are considered as a key element in improving the quality of prescriptions and in preventing ADEs among patient. There are three types of interventions: 1) prescription analysis, 2) adherence evaluation and 3) clinical medication review. The clinical medication review is defined as a structured, critical examination of a patient’s drug prescription with the objectives of reaching an agreement with the patient about their treatment, optimizing the impact of drugs, minimizing the number of drug related side effects rationalizing and simplifying drug regimens and reducing the iatrogeny. The clinical medication review included five interventions: medication history, review of current medication, pharmaceutical intervention, multidisciplinary review of drug prescriptions and medication liaison service (comprehension medication history, discharge letter transmitted to referent physician (RP) of the patient and community pharmacist, discharge counseling). Previous studies have found a positive impact of these interventions on the reduction of PIP, polymedication, adverse drug events in hospitalized elderly and in primary care [1, 30–32]. However, impact of such interventions by the clinical pharmacist integrated in a multidisciplinary approach has not been evaluated in terms of evolution of functional autonomy [33]. The evolution towards functional disabilities, frequent with aging, is at the heart of the concerns of public health authorities. Due to an increasing proportion of elderly in the population and the increase in life expectancy, the care-dependent patients increase the human and financial costs [34]. The evolution of loss of functional autonomy has many causes and risk factors, among which some could be prevented. Among these causes or risk factors, cardiovascular disease, stroke, type 1 or 2 diabetes, impairment of cognitive performance, sleep disorders, depression, a recent hospitalization and polypharmacy may have negative impact on functional autonomy [35, 36]. These observations from the literature make us assume the existence of an indirect link between inappropriate drug prescriptions and accelerated evolution to functional dependence, through suboptimal treatment of chronic diseases and greater frequency of adverse events. A literature review showed that the inappropriate prescription of drugs has a negative impact on functional status of patients; the most offending drugs are benzodiazepines, and anticholinergic drugs [37].

By intervening at the drug prescription, it appears that a profit could be achieved in terms of slowing the progression towards functional dependency. Thus, the improvement of drug prescriptions could delay or prevent the loss of functional autonomy by reducing the risk of ADE, such as falls or cognitive decline and improving the management of chronic diseases. Our hypothesis is that an optimization program of the drug prescribing may slow progression to functional dependence.

## Methods/design

### Aims

#### Primary aim

The primary aim of the OPTIM study is to assess the impact of an optimization program of drug prescribing consisting in a clinical medication review by a pharmacist on the evolution of functional autonomy level, measured during 18 months of follow-up.

Secondary aims

1. In patients, to evaluate the impact of the optimization program of drug prescribing at 18 months of follow-up on: risk of all-cause hospital admission, risk of all-cause emergency department visit, risk of onset of institutionalization, risk of falls, the overall cognitive function and its evolution, the quality of life, anxiety and depressive disorders, drug adherence, pain, and the risk of all-cause death.
2. Among the RP of patients, to evaluate the impact of the optimization program of drug prescribing at 18 months of follow-up, on: PIP, drug related problem (DRP) identified by the pharmacist, the RP acceptance of pharmacist’s interventions (PI).

### OPTIM trial design

The OPTIM study is a multicenter Randomized Controlled Trial (RCT) with two parallel groups (1-intervention, 2-control), and with an exploratory framework.

### Study setting

The study population will include elderly outpatients visiting for the first time one of the two centers included in the study: the geriatric and memory center of an academic hospital (Charpennes Hospital, University Hospital of Lyon, Villeurbanne, France) and the geriatric and memory center of a non-academic hospital, (Mont d’Or Hospital, Albigny-sur-Saône, France).

### Participants

The inclusion criteria of patients are: Patients aged 65 and over, patients received for the first time in a geriatric or memory consultation, patients living at home, patients with the ability to express themselves orally or in writing in French sufficiently to carry out clinical assessments, patients who led the last drugs prescription from his referring physician, at the geriatric/memory consultation (in current practice, patients should take the last prescription established by the referring physician), and patients accompanied by a caregiver.

Exclusion criteria include: Patients with no discernment and patient put under legal protection.

### Interventions

The intervention group will participate to the optimization program: clinical medication review performed by a pharmacist in cooperation with the clinician. This aim is to identify actual and potential DRP, to decrease the potential iatrogeny of drug prescription and to improve the drug adherence of the patient. This intervention will be standardized across participating centers through a “Drug prescription optimization” form. The pharmacist will complete this report form including the patient data (medical, social, lab results and medication), their synthesis of medication review, and their PI in order to achieve drug optimization and their counseling/specific strategies in order to improve the drug adherence. In our study, the clinical medication review will be at the inclusion, 6 months and 18 months.

The clinical medication review will encompass various steps:

- Medication historic, which aims to obtain an as possible complete and accurate list of patient’s previous and current home prescriptions and over-the-counter medications. This should not merely list the medications prescribed or dispensed to the patient, but should include “as-required” medicines and all medications that the patient is currently taking. It will be performed using all available sources of information including previous inpatient discharge summary, community pharmacist and RP records. For each patient, the pharmacist will collect the medications comprehensive list including the name, dose, frequency and route of administration of each medication.
- Review of current medication and Pharmacists’ intervention

The review of current medication performed by the pharmacist, in collaboration with the clinician (specialist physician), will also identify DRP (including pharmacological redundancy, medication overdose, need for a change in dosage form and PIP) taking into account the specificities of drug management in elderly patients. The DRP will be identified through a structured approach for each patient and the pharmacist will perform PI. The medication review will be standardized through various tools, including current national professional guidelines and international recommendations, medications databases, and prescription appropriateness as assessed by a set of validated quality indicators including Screening Tool of Older Persons’ potentially inappropriate prescriptions and Screening Tool to Alert doctors to Right Treatment (STOPP-START) and Beers criteria.

The PI are defined as “any action initiated by a pharmacist directly resulting in a change of the patient’s management or therapy’ to the physician” and including addition of a new drug, discontinuation, switch, dose adjustment, optimization of administration and drug monitoring.

In order to optimize drug adherence, the pharmacist will provide comprehensive counseling and perform specific adherence strategies (information about medications and administration).

- Multidisciplinary revision of drug prescription and medication liaison service: at the end, the standardized report form containing the medication review synthesis, details of the DRP and the PI will be provided by the pharmacist to the medical specialist. If they agree, a discharge letter with medication review synthesis, details of the DRP and the PI will transmit to the patient’s RP. Notices will be sent only to referring physicians of patients, who can apply or not the recommendations.

The control group will receive the current management of outpatients in geriatric or memory consultation, during which the intervention of a clinical pharmacist is not provided. There will be a history of the drugs prescribing leading to pharmaceutical recommendations by the clinical pharmacist, but the discharge letter with medication review synthesis, details of the DRP and the PI will not be transmitted to the RP and community pharmacist.

### Outcomes

The primary outcome will be the evolution of patients’ functional autonomy level assessed with two scales: The Instrumental Activities of Daily Living (IADL) scale of Lawton and the 6-item Disability Assessment of Dementia (DAD-6) scale. Each scale will be analyzed separately. The evolution of functional autonomy will be calculated using the successive evaluations of the scales. The IADL scale assesses the level of functional autonomy of a patient through the assessment of instrumental activities of daily living including ability to use the telephone, transportation, shopping, managing medications, manage a budget, prepare meals, maintain the house and do the laundry. The rating scale provides a score from 0 to 8. A higher score indicates a higher level of dependency, while a lower score reflects a lower level of dependence. The IADL scale consists of 8 questions. The DAD-6 assesses the patient’s activities in his daily life. It includes six questions assessing the degree of autonomy for the following activities: Food, use the telephone or the computer, moving outside, finance and correspondence, medications, leisure and home maintenance. The score ranges from 0 to 18 points, the higher the score is, the more the patient is autonomous. The IADL and DAD-6 scales will be collected during an interview between the patient’s primary caregiver and a neuropsychologist, a nurse or a physician.

The secondary outcomes collected for patients will be the occurrence and the number of all-cause hospitalizations and all-cause emergency department visit after inclusion, admission in institution, and the delay before institutionalization, the occurrence and number of falls after inclusion, the overall cognitive function estimated by the Mini Mental Sate Examination (MMSE) [38] at baseline and its evolution assessed using the successive measures performed at each visit, the quality of life of the patients measured by the questionnaire EUROQOL 5D (EQ5D) [39], the depression and anxiety disorders measured respectively with the mini-GDS scale [40], and the Hamilton scale [41], the compliance of patients with treatment measured with the Girerd questionnaire [42], pain measured with an ordinal scale from 0 to 10, and the possible occurrence of all-cause death. The secondary outcomes collected for RP will be the number of PIP after intervention, and the type of DRP.

### Participant timeline

The OPTIM study is part of the routine care of patients at geriatric/memory center. Four successive evaluations are planned for the participants (Table 1). The inclusion is planned at the first consultation of the patients at the geriatric/memory center. Three others evaluations during follow-up are planned: at 1 month and after 6 months and 18 months. At 6 and 18 months, the patients will undergo a consultation at the geriatric/memory center, whereas the limited evaluation at 1 month will be performed by phone with the primary caregiver.

**Table 1:** Schedule of enrolment, intervention and assessments of the OPTIM study

	Study Period				
	Enrolment	Allocation	Follow-up		
Timepoint	- *t1*	0	*t*_*1*_ *: 1 month*	*t*_*2*_: *6 months*	*t*_*3*_: *18 months - Close-out*
Enrolment:					
Eligibility screen	X				
Information to patients/careviger	X				
Collection of non-opposition to participate	X				
Randomized allocation		X			
Intervention:					
*(1) Intervention: Optimization of drug prescribing*		X	X	X	
*(2) Control*					
Assessments:					
*Collected in patient record:* * Sociodemographic data * Diagnosis and stage * Current comorbidities, history of comorbidities, lifestyle habits * Biological exam and artrial pressure when available		X			
*Primary outcomes:* * IADL scale * DAD-6 scale		X	X	X	X
*Secondary outcomes:* * MMSE (cognitive function) * mini-GDS (depression) * Hamilton scale (anxiety) * Pain scale * EUROQOL-5D (quality of life) * Girerd scale (compliance to drug prescribing) * Event and date of event: hospitalization, falls, recourse of emergency, institutionalization, death		X		X	X
Drugs prescribing		X	X	X	X

### Sample size

The number of subjects needed was calculated on the primary outcome (IADL) to demonstrate a significant difference in the evolution of functional activities between the intervention group and the control group. The IADL score in patients aged 70 years deteriorates on average of 16% over a period of 18 months. This evolution would represent a change of 1.3 percentage points (SD 2) expected in the control group in our study population. To highlight a 20% difference of the evolution of this score, equivalent to a change of 1.04 points of IADL in the intervention group, for an alpha = 0.05 and a power of 80% (two-tailed test), it is necessary to include 224 patients (112 per group). A first observation made in the Charpennes Hospital showed that RP referred 2 patients in average at the memory center. Taking the conservative assumption of 4 patients on average per referent physician, the inflation factor applied was 1.03 for an intra-class coefficient (ICC) of 0.01 [43, 44]. Taking into account the ICC, the number of subjects needed was 232 patients spread between the 2 study groups. Finally, the number of subjects required was corrected for an expected attrition rate of 25%; the corresponding correction factor was 1.3. Assuming that the rate is the same in the 2 groups, the number of subjects required was 302 patients.

### Recruitment

Prior estimations of the number of patients visiting the recruiting centers show the capacity of inclusion of the number of subjects needed over 12 months. Several strategies are envisaged to follow up the study population and allow the collection of data. In case the patient does not show up to the consultation of follow-up, the patient will be contacted to propose a new visit or to collect the reason of absence to the consultation.

### Assignment of interventions

#### Randomization

Random allocation is stratified by center [45]. Patients are assigned to intervention or control group in the study after the eligibility criteria are met. The randomization is performed by following a list of random allocation generated by computer, by the project manager. The unit of randomization is then the patient since the enrollment in the study begins with the patient. However, to limit the risk of contamination due to the fact that a RP can have several patients included in the study, patients with the same RP will be allocated to the same study group. Specifically, each patient coming in geriatric or memory consultation and whose the RP was not identified for a patient previously included, will be assigned randomly to one of the study groups: intervention, or control. Patient will be enrolled by the geriatric/neurologist doctor during the consultation. When a new patient has the same RP that a patient previously enrolled in the study, this new patient will automatically be assigned to the same study group as the previous patient. Finally, the number of patients per RP will be limited to 4.

#### Blinding

The study is considered as open-label. The PI will perform the clinical medical review for all patients included in the study, regardless of their allocation group. Patients and RP will be aware of group allocation by the nature of the intervention which may lead to changes in their drug prescriptions. However, as the primary outcome is evaluated among the patient and by its nature, we assume that the knowledge by patients of their allocation group in the study will have little or no impact on the evolution this outcome.

### Data collection, management, and statistical analysis

#### Data collection methods and data management

The clinical data are collected in a case report form (CRF) assigned to each patient, referred by a unique numeric identifier, using the patient medical record and during the consultation, by a trained neuropsychologist or nurse. Primary and secondary outcomes are collected during the assessment at the consultation. Data are then entered into a computerized database by the neuropsychologist or the nurse. Drugs are collected by the clinical pharmacist using the RP prescriptions and those of the specialist physician at hospital after the consultation. Drugs data and pharmaceutical analysis and PI are entered in the computerized database by the clinical pharmacist. The data collected in the CRF and in the database will be verified for accuracy, missing data, and data consistency with the documents ‘source’ (medical records, visits schedules). Data quality will be analyzed during the study. The completeness and the information will be checked for plausibility in each case report form by a clinical research associate (CRA).

#### Statistical analysis

The analyzes will be performed with an intention-to-treat principle, both RP-patients will remain in their group allocation determined by the randomization procedure, regardless of the nature of the intervention that may have been applied during the study. Analysis per protocol will be performed to verify whether the results are similar with the two approaches. The presence of missing data and extreme values will be verified [46]. Missing data on the primary outcome may be taken into account to some extent in the statistical models used. The impact of missing data on the results will be evaluated as appropriate.Statistical analyzes will be performed using SPSS software (version 19.0, SPSS Inc. statistics). The analyses will focus on data at the patient level and statistical models will take into account possible correlations between data, such as the RP who can be considered as a hierarchical level above the patient unit [47]. The choice of tests will also be adapted to the nature of the variables, and based on the assumptions for the application testing. The significance level for the tests performed on the primary outcome will be 0.05. This threshold will be adjusted for multiple comparisons and sub-analyzes of secondary outcomes when appropriate (Scheffe, Bonferoni correction) [48]. The tests will be bilateral.

#### Descriptive analyzes

A flow-chart will be presented to describe the number of RP per study group, the number of patients selected at inclusion, and the number of patients being followed according to the terms of the protocol, and the loss of follow-up.

Patient characteristics will be compared between the participating centers. Patient characteristics will be compared between the included and followed patients in the study and patients loss of follow-up to assess possible bias. Patient characteristics at baseline will be described for the entire study population, and study group. Quantitative variables will be summarized by mean and standard deviation or median and percentiles if their distribution is not symmetrical. Categorical variables will be presented by frequencies and numbers.Comparisons will be made to test patient characteristics at baseline between the two allocation groups. Means will be compared using the Student *t* test or Mann-Whitney U as appropriate. The proportions will be compared using the chi-square test of two Pearson. The tests will be adjusted to the design effect of the study [49, 50].

#### Comparative analyzes to assess the impact of the intervention on the primary outcome

The ICC will be calculated on the primary outcome for the entire population and by groups to verify that the correlation between observations is similar between groups. The effect of the intervention on evolution of the level of autonomy function will be estimated in a generalized linear mixed model. The mixed model will account for both fixed and random effects, hierarchy data (patient characteristics correlated between them for a same RP) and repeated measurements of the primary outcome [51, 52]. The evolution of the primary outcome will be defined as the dependent variable, explained by the effect of the study group, the time effect and the interaction between these two effects. The intervention will be considered significant if the interaction between time and the study group is significant. The significance level of each term in the model will be presented. The coefficients associated with variables in the model, their 95% confidence interval and significance level will be presented. The effects of covariates will be tested in the model: recruiting center, age, sex, comorbidities, education level and estimates will be adjusted if the effects are significant to account for these confounders. The interaction between patient characteristics and study group will be tested one by one, and analyzes will be stratified if the interactions are significant and that the result is considered clinically relevant.

The impact of the intervention on the secondary outcomes will be investigated using generalized linear mixed models. To explain the dependent variables, other models such as nonlinear mixed effects models or Cox model will be used [53].

#### Data monitoring

The CRA will ensure the successful completion of the study, and the collection of data. The CRA will also ensure the compliance with the study protocol, and the organization of the follow-up of the patients.

#### Confidentiality

The nominative information of the patients enabling to conduct the follow up of the study will be kept in a separate file that does not contain their clinical data and whose access will be protected by a password. The primary investigator and the persons involved in the conduct of the research will ensure the protection of the confidentiality of the data.

#### Harms – End of protocol

Patients will be excluded from the follow-up of study if they no longer wish to participate in the study at any time of the conduct of the study. Information on the temporary or permanent cessation of the participation of a patient will be collected in the patient file. The previously collected data before exclusion may however be used as part of the study, as stated in the information letter. If a visit is missing during the follow-up of the study, but the patient did not indicate that he would not participate to the study, the patient will still be included in the study for the next follow-up.

#### Protocol amendments

In case of important protocol modifications, new ethical board approvals are required and will be asked. These modifications would then be communicated in future publications.

#### Dissemination policy

The results of the primary objective will be published in a peer-reviewed journal. Separate manuscripts may be published on the secondary objectives. All authors of future publications will have to meet the criteria for authorship stated in the Uniform Requirements for Manuscripts Submitted to Biomedical Journals by the International Committee of Medical Journal Editors.

## Discussion

The implementation of the “OPTIM” program should enable optimization of drug prescribing in elderly patients and therefore slow or prevent progression to loss of functional autonomy. It should also help to develop and strengthen collaboration and communication between the team of geriatric/neurologist at the hospital consultation, the pharmacist and the RP who are all involved in caring for the patient’s health. The benefits for the patient are thus optimizing its medical management by linking health professionals met during his care pathway. In addition, pharmaceutical recommendations sent to RP should help raise awareness of the prescription of drugs in these patients.

Some limits are already considered. In some cases (allergy, occurrence of adverse effects), the substitution of some potentially harmful drugs could not be achieved. Furthermore, some patients may experience a reluctance to change the medication that they used to follow for many years. In any case, the drug outpatient prescription must remain an essential aspect of the relationship between the patient and his RP. The lack of blinding due to the nature of the intervention may be responsible of a bias; however the knowledge by patients of their allocation group may have little or no impact on the evolution their functional autonomy level.

The study is implemented in the current care of patients followed at geriatric and memory consultation at the hospital and should allow to assess its impact in everyday practice. If the OPTIM study demonstrates a positive impact on older patients, implementing sustainable way of drug therapy optimization program could be considered on other hospitals in connection with their pharmacy service.

## Abbreviations

ADE: Adverse drug events
CRA: Clinical research associate
CRF: Case report form
DAD-6: 6-item Disability assessment of dementia
DRP: Drug related problem
IADL: Instrumental activities of daily living
ICC: Intra-class coefficient
MMSE: Mini mental sate examination
PI: Pharmacist’s interventions
PIP: Potentially inappropriate prescriptions
RP: Referent physician
